# Supplementary material for: The Gut Bacteria Dysbiosis Contributes to Chronic Graft-Versus-Host Disease Associated With a Treg/Th1 Ratio Imbalance
Source: Front Microbiol. 2022 Sep 8;13:813576. doi: 10.3389/fmicb.2022.813576 (PMC9493085; doi:10.3389/fmicb.2022.813576)
Supplement: Supplementary file 1 [file Data_Sheet_1.zip › P101SC18090073-01-B1-3-4_result/ReadMe.html]

| result |  |
| --- | --- |
| \|-- 00.RawData | 【下机数据进行低质量碱基过滤后的数据】 |
| \| \|-- Sample\_Name | 【每个样品对应的原始下机数据和初步质控后的数据】 |
| \| \| \|-- \*.fq.gz | 【reads 去除 baraode 和 primer 后得到的FASTQ格式序列】 |
| \| \| \|-- .fna | 【reads 去除 baraode 和 primer 后得到的FASTA格式序列】 |
| \|-- 01.CleanData | 【质控后可用于后续分析的有效数据】 |
| \| \|-- QCstat.xls | 【数据预处理统计及质控信息表】 |
| \| \|-- Sample\_Name | 【每个样品的质控结果】 |
| \| \| \|-- *.fna(*.fna) | 【质控后序列的 FASTA 格式文件】 |
| \| \| \|-- histograms.xls | 【质控后的序列长度分布统计表】 |
| \|-- 02.OTUanalysis | 【OTUs 聚类和物种注释结果】 |
| \| \|-- genus\_evolutionary\_tree | 【属水平物种进化树圈图】 |
| \| \|-- OTUs.fasta | 【OTUs 代表序列】 |
| \| \|-- OTUs.tax\_assignments.txt | 【OTUs 物种注释结果】 |
| \| \|-- OTUs.tre | 【OTUs 进化树文件】 |
| \| \|-- otu\_table\_even.biom | 【均一化处理后 biom 格式的绝对丰度表】 |
| \| \|-- taxa\_abundance | 【每个样品中物种丰度信息】 |
| \| \| \|-- Absolute | 【OTUs 绝对丰度】 |
| \| \| \| \|-- otu\_table.\*.absolute.xls | 【界门纲目科属种（k,p,c,o,f,g,s）水平上的绝对丰度】 |
| \| \| \| \|-- otu\_table.txt | 【OTUs 绝对丰度表】 |
| \| \| \|-- Evenabs | 【均一化处理后的绝对丰度（均一化处理使每个样品的 OTUs 丰度之和相等）】 |
| \| \| \| \|-- otu\_table.absolute.xls | 【OTUs 绝对丰度】 |
| \| \| \| \|-- otu\_table.\*.absolute.xls | 【界门纲目科属种（k,p,c,o,f,g,s）水平上的绝对丰度】 |
| \| \| \|-- Relative | 【均一化处理后的相对丰度（均一化处理使每个样品的 OTUs 丰度之和相等）】 |
| \| \| \| \|-- otu\_table.\*.relative.xls | 【界门纲目科属种（k,p,c,o,f,g,s）水平上的相对丰度】 |
| \| \| \| \|-- otu\_table.relative.xls | 【OTUs 相对丰度】 |
| \| \| \|-- Relative\_group | 【组OTUs 相对丰度】 |
| \| \| \| \|-- otu\_table\_group.\*.relative.xls | 【组间界门纲目科属种（k,p,c,o,f,g,s）水平上的相对丰度】 |
| \| \| \| \|-- otu\_table\_group.relative.xls | 【组间OTUs 相对丰度】 |
| \| \|-- taxa\_heatmap | 【物种注释聚类热图】 |
| \| \| \|-- cluster | 【门纲目科属（p,c,o,f,g）水平上的物种聚类热图】 |
| \| \| \| \|-- cluster\*.{pdf,png,txt} | 【门纲目科属（p,c,o,f,g）水平上的物种聚类热图和作图数据】 |
| \| \| \|-- cluster\_group | 【门纲目科属（p,c,o,f,g）水平上的物种聚类热图】 |
| \| \| \| \|-- cluster\*.{pdf,png,txt} | 【门纲目科属（p,c,o,f,g）水平上的分组物种聚类热图和作图数据】 |
| \| \| \|-- OTU\_heatmap | 【OTUs 注释和丰度热图】 |
| \| \|-- taxa\_stat | 【OTUs 分析统计结果】 |
| \| \| \|-- Classified\_stat.{png,svg} | 【注释到界门纲目科属种（k,p,c,o,f,g,s）水平上的 Tags 数目分布图】 |
| \| \| \|-- classified\_stat.xls | 【注释到界门纲目科属种（k,p,c,o,f,g,s）水平上的 Tags 数目统计表】 |
| \| \| \|-- Sample\_Tags-OTUs\_dis.png | 【各样品的 Tags 及 OTUs 数目统计分布图】 |
| \| \| \|-- Tags\_stat.xls | 【各样品的 Tags 及 OTUs 数目统计表】 |
| \| \|-- taxa\_tree | 【物种分类树】 |
| \| \| \|-- \*.taxtree.png | 【单个样品的物种分类树】 |
| \| \| \|-- all.taxtree.png | 【所有样品的物种分类树】 |
| \| \|-- taxa\_tree\_group | 【所有分组的物种分类树】 |
| \| \| \|-- all.taxtree.svg | 【所有样本不同比例的物种分类树】 |
| \| \| \|-- \*.taxtree.svg | 【单个样品的物种分类树】 |
| \| \|-- ternaryplot(\_group) | 【ternaryplot 分析结果】 |
| \| \| \|-- Sample\_Name | 【三元相图】 |
| \| \|-- top10 | 【各个样本门纲目科属（p,c,o,f,g,s）水平上的 top10 物种相对丰度柱形图以及属水平的top30物种相对丰度柱形图】 |
| \| \|-- top10\_group | 【各分组门纲目科属（p,c,o,f,g,s）水平上的 top10 物种相对丰度柱形图以及属水平的top30物种相对丰度柱形图】 |
| \| \|-- Funnction prediction | 【功能预测】 |
| \| \| \|-- FUNGuild | 【FUNGuild功能预测】 |
| \| \| \| \|--  00.annotation | 【预测结果统计】 |
| \| \| \| \|--  01.barplot | 【预测结果柱形图展示】 |
| \| \| \| \|--  02.heatmap | 【预测结果热图展示】 |
| \| \| \| \|--  03.PCA | 【PCA结果】 |
| \| \| \|-- FAPROTAX | 【FAPROTAX 功能预测】 |
| \| \| \| \|--  00.annotation | 【预测结果统计】 |
| \| \| \| \|--  01.barplot | 【预测结果柱形图展示】 |
| \| \| \| \|-- 02.heatmap | 【预测结果热图展示】 |
| \| \| \| \|-- 03.PCA | 【PCA结果】 |
| \| \| \|-- Tax4Fun | 【Tax4Fun功能预测】 |
| \| \| \| \|-- 00.Predicted\_RawData | 【预测结果统计】 |
| \| \| \| \|--  01.Relative | 【预测结果相对丰度】 |
| \| \| \| \|-- 02.Top10\_bar\_plot | 【预测结果top10柱形图展示】 |
| \| \| \| \|-- 03.Cluster\_heatmap | 【预测结果热图展示】 |
| \| \| \| \|--  04.PCA | 【PCA】 |
| \| \| \| \|-- 05.Venn\_flower | 【venn图】 |
| \| \| \| \|-- 06.iPATH | 【代谢通路结果】 |
| \| \| \|-- PICRUSt | 【PICRUSt功能预测】 |
| \| \| \| \|-- 00.Predicted\_RawData | 【预测结果统计】 |
| \| \| \| \|--  01.Absolute | 【预测结果绝对丰度】 |
| \| \| \| \|--  02.Relative | 【预测结果相对丰度】 |
| \| \| \| \|-- 03.Top10\_bar\_plot | 【预测结果top10柱形图展示】 |
| \| \| \| \|-- 04.Cluster\_heatmap | 【预测结果热图展示】 |
| \| \| \| \|--  05.PCA | 【PCA】 |
| \| \| \| \|-- 06.Venn\_flower | 【venn图】 |
| \| \| \| \|-- 07.iPATH | 【代谢通路结果】 |
| \| \| \|-- Bugbase | 【Bugbase表型预测】 |
| \| \| \| \|-- normalized\_otus | 【标准化 OTU 表】 |
| \| \| \| \|-- otu\_contributions | 【表型预测结果堆叠柱状图】 |
| \| \| \| \|-- predicted\_phenotypes | 【表型预测结果比较箱线图】 |
| \| \| \| \|-- thresholds | 【阈值选择表】 |
| \|-- 03.AlphaDiversity | 【alpha 多样性分析结果】 |
| \| \|-- Alpha\_div | 【组间 Alpha 多样性比较箱型图】 |
| \| \| \|-- ACE | 【ACE指数】 |
| \| \| \| \|-- ACE.png | 【ACE 指数箱型图】 |
| \| \| \| \|-- ACE\_Tukey.txt | 【ACE 指数多组间方差分析】 |
| \| \| \| \|-- ACE\_wilcox.txt | 【ACE 指数多组间非参数 wilcox 检验】 |
| \| \| \|-- chao1 | 【chao1指数】 |
| \| \| \| \|-- chao1.png | 【chao1 指数箱型图】 |
| \| \| \| \|-- chao1\_Tukey.txt | 【chao1 指数多组间方差分析】 |
| \| \| \| \|-- chao1\_wilcox.txt | 【chao1 指数多组间非参数 wilcox 检验】 |
| \| \| \|-- goods\_coverage | 【覆盖率】 |
| \| \| \| \|-- goods\_coverage.png | 【goods\_coverage 指数箱型图】 |
| \| \| \| \|-- goods\_coverage\_Tukey.txt | 【goods\_coverage 指数多组间方差分析】 |
| \| \| \| \|-- goods\_coverage\_wilcox.txt | 【goods\_coverage 指数多组间非参数 wilcox 检验】 |
| \| \| \|-- observed\_species | 【observed\_specie 指数箱型图】 |
| \| \| \| \|-- observed\_species\_Tukey.txt | 【observed\_species 指数多组间方差分析】 |
| \| \| \| \|-- observed\_species\_wilcox.txt | 【observed\_species 指数多组间非参数 wilcox 检验】 |
| \| \| \|-- PD\_whole\_tree | 【PD\_whole\_tree】 |
| \| \| \| \|-- PD\_whole\_tree.png | 【PD\_whole\_tree箱型图】 |
| \| \| \| \|-- PD\_whole\_tree\_Tukey.txt | 【PD\_whole\_tree Turkey检验】 |
| \| \| \| \|-- PD\_whole\_tree\_wilcox.txt | 【PD\_whole\_tree wilcox检验】 |
| \| \| \|-- Alpha\_div.pdf | 【非参数检验文件说明】 |
| \| \| \|-- shannon | 【shannon指数】 |
| \| \| \| \|-- shannon.png | 【shannon 指数箱型图】 |
| \| \| \| \|-- shannon\_Tukey.txt | 【shannon 指数多组间方差分析】 |
| \| \| \| \|-- shannon\_wilcox.txt | 【shannon 指数多组间非参数 wilcox 检验】 |
| \| \| \|-- simpson | 【simpson指数】 |
| \| \| \| \|-- simpson.{png} | 【simpson 指数箱型图】 |
| \| \| \| \|-- simpson\_Tukey.txt | 【simpson 指数多组间方差分析】 |
| \| \| \| \|-- simpson\_wilcox.txt | 【simpson 指数多组间非参数 wilcox 检验】 |
| \| \| \|-- beewarm | 【beewarm】 |
| \| \|-- alpha\_diversity\_index\_group.txt | 【组间α多样性指数】 |
| \| \|-- alpha\_diversity\_index.txt | 【样本α多样性指数】 |
| \| \|-- venn\_figure/ | 【 基于OTU的Venn图】 |
| \| \| \|-- vennrdata | 【 venn图数据】 |
| \| \| \| \|-- 1.\*.uniq.otu.xls | 【 各样本独有OTU信息】 |
| \| \| \|-- venn\_display.png | 【 venn图展示】 |
| \| \|-- Flower\_figure | 【 基于OTU的花瓣图】 |
| \| \| \|-- flowerdata | 【 花瓣图数据】 |
| \| \| \| \|-- 1.\*.uniq.otu.xls | 【 各样本独有OTU信息】 |
| \| \| \|-- flower\_display.png | 【 花瓣图展示】 |
| \| \|-- group\_observed\_species.png | 【 分组物种数目饱和度曲线】 |
| \| \|-- group\_rank\_abundance.png | 【分组Rank Abundance 曲线】 |
| \| \|-- observed\_species.png | 【 样本物种数目饱和度曲线】 |
| \| \|-- plot\_observed\_species.xls | 【 样本物种数目饱和度曲线数据文件】 |
| \| \|-- rank\_abundance.png | 【 样本Rank Abundance 曲线】 |
| \| \|-- Specaccum | 【物种累积曲线】 |
| \| \|-- specaccum\_test.png | 【物种累积曲线图】 |
| \|-- 04.BetaDiversity | 【Beta 多样性分析结果】 |
| \| \|-- Adonis | 【Adonis 分析结果】 |
| \| \| \|-- bray\_adonis.txt |  |
| \| \|-- Amova | 【Amova 分析结果】 |
| \| \| \|-- (un)weighted\_unifrac\_amova.txt |  |
| \| \|-- Anosim | 【Anosim 分析结果】 |
| \| \| \|-- stat\_anosim.txt |  |
| \| \| \|-- \*.{pdf,png} | 【Anosim 分析箱图结果】 |
| \| \|-- Beta\_div | 【组间 Beta 多样性比较箱型图】 |
| \| \| \|-- Beta\_div.pdf | 【非参数检验文件说明】 |
| \| \| \|-- weighted\_unifrac.{png,pdf} | 【基于加权的 unifrac 距离的 Beta 多样性箱型图】 |
| \| \| \|-- weighted\_unifrac\_TukeyHSD.txt | 【基于加权的 unifrac 距离的多组间方差分析】 |
| \| \| \|-- weighted\_unifrac\_wilcox.txt | 【基于加权的 unifrac 距离的多组间非参数 wilcox 检验】 |
| \| \| \|-- unweighted\_unifrac.{png,pdf} | 【基于非加权 unifrac 距离的 Beta 多样性箱型图】 |
| \| \| \|-- unweighted\_unifrac\_TukeyHSD.txt | 【基于非加权的 unifrac 距离的多组间方差分析】 |
| \| \| \|-- un weighted\_unifrac\_wilcox.txt | 【基于非加权的 unifrac 距离的多组间非参数 wilcox 检验】 |
| \| \|-- beta\_div\_heatmap | 【样品unifrac 距离热图】 |
| \| \| \|-- beta\_diversity.heatmap.png | 【包含两种距离的热图】 |
| \| \| \|-- beta\_diversity.heatmap.svg |  |
| \| \| \|-- beta\_diversity.heatmap.UnW.png | 【非加权 unifrac 距离热图】 |
| \| \| \|-- beta\_diversity.heatmap.UnW.svg |  |
| \| \| \|-- beta\_diversity.heatmap.W.png | 【加权 unifrac 距离热图】 |
| \| \| \|-- beta\_diversity.heatmap.W.svg |  |
| \| \| \|-- unweighted\_unifrac\_sorted\_otu\_table.txt | 【非加权 unifrac 距离值】 |
| \| \| \|-- weighted\_unifrac\_sorted\_otu\_table.txt | 【加权 unifrac 距离值】 |
| \| \|-- beta\_div\_heatmap\_group | 【组间unifrac 距离热图】 |
| \| \| \|-- beta\_diversity.heatmap.png | 【包含两种距离的热图】 |
| \| \| \|-- beta\_diversity.heatmap.svg |  |
| \| \| \|-- beta\_diversity.heatmap.UnW.png | 【非加权 unifrac 距离热图】 |
| \| \| \|-- beta\_diversity.heatmap.UnW.svg |  |
| \| \| \|-- beta\_diversity.heatmap.W.png | 【加权 unifrac 距离热图】 |
| \| \| \|-- beta\_diversity.heatmap.W.svg |  |
| \| \| \|-- unweighted\_unifrac\_Description\_otu\_table.txt | 【非加权 unifrac 距离值】 |
| \| \| \|-- weighted\_unifrac\_Description\_otu\_table.txt | 【加权 unifrac 距离值】 |
| \| \|-- Environmen\_factor | 【环境因子分析】 |
| \| \| \|-- mantel\_test | 【门纲目科属种以及otu（p,c,o,f,g,s,otu）水平上的mantel\_test 分析结果】 |
| \| \| \|-- multiCCA | 【multiCCA 分析结果】 |
| \| \| \| \|-- BioENV.txt | 【Bioenv筛选后得到的环境因子组合】 |
| \| \| \| \|-- CCA | 【包含所有环境因子的CCA和RDA】 |
| \| \| \| \|-- CCA\_BioENV | 【经Bioenv筛选后环境因子的CCA和RDA】 |
| \| \| \| \|-- CCA\_envfit | 【经envfit筛选后环境因子的CCA和RDA】 |
| \| \| \| \|-- CCA\_VIF | 【经VIF筛选后环境因子的CCA和RDA】 |
| \| \| \| \|-- VIF.txt | 【VIF筛选后得到的环境因子组合】 |
| \| \| \|-- spearman | 【门纲目科属种和otu以及alpha（p,c,o,f,g,s,otu,alpha）水平上的 spearman 分析结果,属水平目录下含有环境因子与丰度的玫瑰图展示】 |
| \| \| \|-- genus\_spearman | 【属水平差异物种相关性】 |
| \| \| \|-- VPA | 【VPA 分析结果】 |
| \| \| \|-- VPA1.png | 【VPA分组环境因子解释率图】 |
| \| \| \|-- biomarkers\_raw\_images | 【各 biomarker 在各组样品中的相对丰度比较图】 |
| \| \|--LEfSe | 【LEfSe 分析结果】 |
| \| \| \|-- LDA.\*.{png,svg,pdf} | 【LDA 值分布柱状图】 |
| \| \| \|-- LDA.\*.res | 【LEfSe 统计结果】 |
| \| \| \|-- LDA.\*.tree.{png,svg,pdf} | 【LEfSe 进化分支图】 |
| \| \|-- MetaStat | 【门纲目科属（p,c,o,f,g）水平上的MetaStat 分析结果，属水平目录下含有差异物种热图】 |
| \| \| \| \|-- \*.test.xls | 【门纲目科属（p,c,o,f,g）水平上 MetaStat 分析结果】 |
| \| \| \| \|-- \*.psig.xls | 【从 MetaStat 分析结果中，筛选出的 p value<=0.05 的信息】 |
| \| \| \| \|-- cluster.\*.diff.{pdf,png,t} | 【具有显著性差异物种的 heatmap 热图分析结果和输入文档】 |
| \| \| \| \|-- \*/boxplot | 【具有显著性差异物种的箱图结果】 |
| \| \| \| \|-- \*/PCA | 【具有显著性差异物种的PCA结果】 |
| \| \|-- MRPP | 【MRPP 分析结果】 |
| \| \| \|-- stat\_mrpp.txt |  |
| \| \|-- Network | 【Network 分析结果】 |
| \| \| \|-- group |  |
| \| \|-- NMDS | 【NMDS 分析结果】 |
| \| \| \|-- NMDS\_2.png | 【未标样品名称的 NMDS 图】 |
| \| \| \|-- NMDS.png | 【NMDS 分析结果】 |
| \| \| \|-- NMDS\_with\_cluster2.png | 【带有置信椭圆未标样品名称的NMDS图】 |
| \| \| \|-- NMDS\_with\_cluster.png | 【带有置信椭圆标注样品名称的NMDS图】 |
| \| \| \|-- NMDS\_scores.txt | 【各样品在前两个主成分轴上的位置坐标】 |
| \| \|-- PCA | 【PCA 分析结果】 |
| \| \| \|-- PCA12\_2.png | 【标有样品名称的 PCA 图】 |
| \| \| \|-- PCA12.png | 【未标样品名称的 PCA 图】 |
| \| \| \|-- PCA12*with\_cluster*2.png | 【带有置信椭圆的 PCA 图】 |
| \| \| \|-- PCA12\_with\_cluster.png | 【带有置信椭圆的 PCA 图】 |
| \| \| \|-- pca.csv | 【PCA作图数据】 |
| \| \|-- PCoA | 【PCoA 分析】 |
| \| \| \|-- unweighted\_unifrac | 【基于非加权 unifrac 距离的 PCoA 分析结果】 |
| \| \| \| \|-- emperor\_pcoa\_plots | 【三维 PCoA 网页版展示】 |
| \| \| \| \|-- PCoA12-2.png | 【标有样品名称 PCoA 图】 |
| \| \| \| \|-- PCoA12.png | 【未标样品名称的 PCoA 图】 |
| \| \| \| \|-- PCoA12\_with\_cluster\_2.png | 【带有置信椭圆未标样品名称 PCoA 图】 |
| \| \| \| \|-- PCoA12\_with\_cluster.png | 【带有置信椭圆标有样品名称的 PCoA 图】 |
| \| \| \| \|-- PCoA.txt | 【各个主成分的分析结果】 |
| \| \| \|-- unweighted\_unifrac\_dm.txt | 【用于 PCoA 分析的 unweighted unifrac 距离矩阵】 |
| \| \| \|-- unweighted\_unifrac\_pc.txt | 【PCoA 分析主成分信息】 |
| \| \| \|-- weighted\_unifrac | 【基于加权 unifrac 距离的 PCoA 分析结果】 |
| \| \| \| \|-- emperor\_pcoa\_plots | 【三维 PCoA 网页版展示】 |
| \| \| \| \|-- PCoA12-2.png | 【标有样品名称 PCoA 图】 |
| \| \| \| \|-- PCoA12.png | 【未标样品名称的 PCoA 图】 |
| \| \| \| \|-- PCoA12\_with\_cluster\_2.png | 【带有置信椭圆未标样品名称 PCoA 图】 |
| \| \| \| \|-- PCoA12\_with\_cluster.png | 【带有置信椭圆标有样品名称的 PCoA 图】 |
| \| \| \| \|-- PCoA.txt | 【各个主成分的分析结果】 |
| \| \| \|-- weighted\_unifrac\_dm.txt | 【用于 PCoA 分析的 weighted unifrac 距离矩阵】 |
| \| \| \|-- weighted\_unifrac\_pc.txt | 【PCoA 分析主成分信息】 |
| \| \|-- Simper | 【界门纲目科属种（k,p,c,o,f,g,s）水平上物种对样本差异贡献度分析（simper分析）】 |
| \| \|-- Tree | 【聚类树】 |
| \| \| \|-- unweighted\_unifrac | 【基于非加权 unifrac 距离的聚类结果】 |
| \| \| \| \|-- sorted\_otu\_table\_upgma.tre | 【基于非加权 unifrac 距离的 UPGMA 聚类树文件，MEGA 软件可以打开】 |
| \| \| \| \|-- unweighted\_unifrac.png | 【基于非加权 unifrac 距离的 UPGMA 聚类树图】 |
| \| \| \| \|-- UPGMA.UnW.tree.png | 【基于非加权 UPGMA 聚类树合并图】 |
| \| \| \|-- weighted\_unifrac | 【基于加权 unifrac 距离的聚类结果】 |
| \| \| \| \|-- sorted\_otu\_table\_upgma.tre | 【基于加权 unifrac 距离的 UPGMA 聚类树文件，MEGA 软件可以打开】 |
| \| \| \| \|-- UPGMA.W.tree.png | 【基于加权 UPGMA 聚类树合并图】 |
| \| \| \| \|-- weighted\_unifrac.png | 【基于加权 unifrac 距离的 UPGMA 聚类树图】 |
| \| \|-- Tree\_group | 【分组后加入门水平物种组成的 UPGMA 聚类树图】 |
| \| \| \|--unweighted\_unifrac | 【各组基于非加权 unifrac 距离的聚类结果】 |
| \| \| \| \|-- Description\_otu\_table\_upgma.tre | 【UPGMA 聚类树文件】 |
| \| \| \| \|-- unweighted\_unifrac.png | 【组间非加权UPGMA 聚类树】 |
| \| \| \| \|-- UPGMA.UnW.tree.png | 【组间非加权UPGMA 聚类树合并图】 |
| \| \| \|-- weighted\_unifrac | 【各分组基于加权 unifrac 距离的聚类结果】 |
| \| \| \|-- Description\_otu\_table\_upgma.tre | 【UPGMA 聚类树文件】 |
| \| \| \|-- UPGMA.W.tree.png | 【加权UPGMA 聚类树合并图】 |
| \| \| \|-- weighted\_unifrac.png | 【加权的UPGMA 聚类树】 |
| \| \|-- t.test\_bar\_plot | 【门纲目科属种（p,c,o,f,g,s）水平上的组间差异显著的物种分析】 |
| \| \| \| \|-- DCS-vs-MW.c.mat | 【两样本t-test数据文件】 |
| \| \| \| \|-- DCS-vs-MW.psig.xls | 【两样本t-test数据结果】 |
| \| \| \| \|-- DCS-vs-MW.psig.xls.png | 【组间差异显著的物种分析条形图】 |
| \| \| \|-- DCS-vs-MW.psig.xls.svg |  |
| \| \| \| \|-- DCS-vs-MW.qsig.xls | 【从组间差异显著的物种分析结果中，筛选出的 p value<=0.05 的信息】 |
| \| \| \| \|-- DCS-vs-MW.test.xls | 【组间差异显著的物种分析结果】 |
| \| \|-- Random\_Forest | 【门纲目科属种（p,c,o,f,g,s）水平上的随机森林】 |
| \|-- 05.WebShow | 【网页版展示内容综合，可交互式操作，同时含使用说明】 |
| \| \|-- alpha\_rarefaction\_plots | 【网页版展示稀疏性曲线图】 |
| \| \| \|-- average\_plots | 【α多样性指数图】 |
| \| \| \|-- average\_tables | 【α多样性指数表】 |
| \| \| \|-- rarefaction\_plots.html | 【稀疏性曲线网页图】 |
| \| \|-- krona | 【网页版Krona交互图】 |
| \| \|-- OTU\_heatmap | 【网页版OUT\_heatmap交互图】 |
| \| \|-- taxa\_charts\_html | 【网页版物种分类层级展示】 |
| \| \| \|-- area\_charts.html | 【注释结果面积图展示】 |
| \| \| \|-- bar\_charts.html | 【注释结果柱形图展示】 |
| \| \| \|-- charts | 【网页展示用到的图片】 |
| \| \| \|-- css | 【网页配置文件】 |
| \| \| \|-- js | 【网页配置文件】 |
| \| \|-- WebShow.pdf |  |
| \|-- ReadMe.html |  |

`Tips:`

- MetaStat、Adonis、MRPP、Anosim、Amova、t.test和LEfSe等组间差异分析，每个分组不少于三个生物学重复时才能做相应的统计检验（若组间无差异物种，则相应的检验结果不展示）。
- Network分析，默认每个分组不少于六个生物学重复时方可出图。

  ​

---

```
```
xxxxxxxxxx
```


```
                           如有疑问请咨询项目销售或运营，我们将竭诚为您服务。感谢您对Novogene的信任！
```
```
